# Aqueous extract‐mediated green synthesis of CuO nanoparticles: Potential anti‐tuberculosis agents

**DOI:** 10.1002/fsn3.4227

**Published:** 2024-05-30

**Authors:** Zohreh Zamanian, Elahe Tajbakhsh, Nazila Arbab Soleimani, AbdolMajid Ghasemian

**Affiliations:** ^1^ Department of Microbiology, Shahrekord Branch Islamic Azad University Shahrekord Iran; ^2^ Department of Microbiology, Damghan Branch Islamic Azad University Damghan Iran; ^3^ Noncommunicable Diseases Research Center Fasa University of Medical Sciences Fasa Iran

**Keywords:** antitubercular, astragalus extract, CuO nanoparticles, garlic extract, green synthesis, synergistic

## Abstract

The emergence of drug‐resistant strains in tuberculosis treatment underscores the urgency for novel therapeutic approaches. This study investigates the anti‐tuberculosis activity of green‐synthesized copper oxide (CuO) nanoparticles (NPs) using garlic and astragalus extracts. The physicochemical characterization of the nanoparticles confirms successful synthesis, followed by assessment of their antibacterial properties and safety profile. Rats infected with *Mycobacterium tuberculosis* are treated with nanocomposites derived from garlic extract at doses of 50 mg/kg and 100 mg/kg body weight. Evaluation includes the analysis of Early secreted antigenic target of 6 kDa (ESAT‐6) expression and confirmation of antibodies through molecular assays. Administration of garlic and nanocomposites demonstrates significant inhibitory effects on tuberculosis progression in rats, validated by safety assessments and antibacterial efficacy. Notably, the 100 mg/kg dosage exhibits pronounced mitigation of tuberculosis‐induced oxidative stress and lung damage. In conclusion, the combined administration of garlic extracts and green‐synthesized nanocomposites shows promising efficacy in reducing tuberculosis infection, highlighting a potential avenue for anti‐tuberculosis interventions.

## Graphical abstract

In sum, the mixture of garlic extract and nanoparticles at 100 mg/kg body weight of rats inhibited tuberculosis infection. It exhibited antibacterial activity and antioxidant properties in lung tissue and prevented lung damage. The results are promising and show that garlic extract and nanoparticles can be utilized to prevent tuberculosis. This study was conducted on rats, and the results cannot be used for humans, a major limitation.

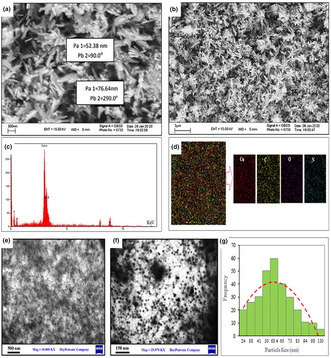

## 1 INTRODUCTION

Infection with *Mycobacterium tuberculosis* has involved one‐fourth of the global population and Tuberculosis (TB) remains a global health concern, with the emergence of drug‐resistant strains posing significant challenges to treatment efforts (Sterling et al., 2020). The disease does not progress to an active form in most patients but can lead to death (Roy Chowdhury et al., 2018). The pathogen can persist in the host in an asymptomatic state of latency (Chee et al., 2018). The size of infectious droplets of *M. tuberculosis* is in the range of 0.65–7.0 μm. Small particles are transmitted into the nasopharyngeal and tracheobronchial regions; however, large particles may become obstructed within the upper airway or oropharynx (Bussi & Gutierrez, 2019). Most patients (90.00%) can respond to *M. tuberculosis* infection by granuloma formation, while other patients start to grow TB germs and latent tuberculosis infection (LTBI) develops into active (Kim & Kim, 2018). Disease control is conducted via decreasing transmission, but this strategy cannot control the disease (Ganmaa et al., 2020). In addition, the lungs have evolved several biological mechanisms to counteract tuberculosis infection, such as responding to oxidative stress (Shastri et al., 2018). It also involves molecular mechanisms. Early secreted antigenic target of 6 kDa (ESAT‐6) is a component of the H56 fusion protein that stimulates the production of interferon‐γ (IFN‐γ) in the QuantiFERON‐TB Gold In‐Tube (QFT‐GIT) interferon‐gamma release assay (IGRA) (Jeremiah et al., 2021). The treatment of *M. tuberculosis* is extremely difficult, owing to a lipid bilayer forming the outer bacterial barrier and the presence of mycolic acids (Lehmann et al., 2018). The treatment of *M. tuberculosis* is based on multidrug chemotherapy, and several drugs are administered to treat tuberculosis, such as rifampicin, isoniazid, pyrazinamide, and ethambutol. Prolonged regimens using the drugs lead to complications, such as drug resistance and the increased emergence of drug‐resistant strains (Lee et al., 2019). Conventional drug regimens often require prolonged administration, leading to increased risks of drug resistance and treatment failure (Lee et al., 2019). Therefore, there is an urgent need for the development of alternative anti‐tuberculosis therapies.

This study focuses on exploring the potential of green‐synthesized copper oxide (CuO) nanoparticles (NPs) derived from garlic and astragalus extracts as novel agents for combating TB infection. Green synthesis offers a sustainable and environmentally friendly approach to nanoparticle production, utilizing plant extracts rich in bioactive compounds as both reducing and stabilizing agents (Abdel‐Daim et al., 2019; El‐Refai, Saleh, et al., 2024; El‐Seedi et al., 2019; Kazemi et al., 2023; Zahin et al., 2020). Garlic and astragalus extracts, known for their antimicrobial properties, serve as promising candidates for nanoparticle synthesis (Abdallah et al., 2023; Mohamed et al., 2021).

Nanoparticles are prepared and produced to treat different diseases and as antibacterial agents (El‐Refai, Mohamed, et al., 2024; Imani & Safaei, 2019; Yousaf et al., 2020). Medicinal plants have been extensively used to treat diseases and nanocomposites' structures (Choodari Gharehpapagh et al., 2021; Grewal et al., 2021; Habibi Zadeh et al., 2020; Mahmoudabadi et al., 2021; Talebi et al., 2021). Garlic (*Allium sativum*) is a medicinal plant that has a pivotal role in maintaining human physiology and is a potential plant to decrease various ailments (Mohamed Ismail et al., 2020). It has been utilized for centuries worldwide by various societies to combat infectious diseases (Kshirsagar et al., 2018). It offers various health advantages, including antibacterial, antioxidant, anticancer, and antiviral properties (Edikresnha et al., 2019; El‐Refai, Mohamed, et al., 2024). The antimicrobial effect of garlic may be ascribed to its organosulfur compounds, commonly referred to as allicin (Sarhan et al., 2016). There have been reports indicating the antibacterial activities of allicin/garlic extract alone and/or in combination with classical antibiotics against *M. tuberculosis* (Dwivedi et al., 2019; Fatima & Prakash Dwivedi, 2020).

On the other hand, metal oxides are utilized for photoconductive, photocatalytic, and photothermal applications (Grewal et al., 2021; Panimalar et al., 2020). They are utilized for an antimicrobial, antifungal agent, and antibiotic activities incorporated into coatings, plastics, and textiles (George et al., 2022; Naika et al., 2015). Copper oxide exhibits distinctive optical, electrical, and magnetic characteristics as a semiconductor metal (Zhang et al., 2014). Copper nanoparticles (CuNPs) prevent the growth of pathogenic bacteria and these applications are used to combat viruses, such as herpes simplex, influenza, and hepatitis A (Alagarasan et al., 2023). On the other hand, with increased concerns about the energy crisis and the challenges of chemical and physical approaches to prepare metal NPs, efforts have been conducted to develop modern alternative chemistry (Cuong et al., 2022). To synthesize safe metal nanoparticles, green synthesis is utilized with the help of plant extracts, such as astragalus extract.

The *Astragalus* genus belongs to the *Fabaceae* family and is extensively found in Europe, North Africa, Asia, and the Mediterranean. Their roots are applied as the primary drug in several countries to increase the immune system and reduce inflammation (Siwicka et al., 2011). It is rich in saponins, phenolics, flavonoids, and polysaccharides (Li et al., 2019) that could be utilized for green synthesis.

The primary goal of this research is to evaluate the efficacy of garlic extract and astragalus extract‐mediated green‐synthesized CuO nanoparticles against TB infection. In addition to assessing their antibacterial activity, it is crucial to characterize the physicochemical properties of the nanoparticles to understand their structure–function relationships and optimize their therapeutic potential. Characterization techniques, such as X‐ray diffraction (XRD), transmission electron microscopy (TEM), and Fourier‐transform infrared spectroscopy (FTIR), will be employed to elucidate the size, morphology, and chemical composition of the nanoparticles.

By integrating the biological activity of the nanoparticles with their physicochemical characterization, this study aims to provide valuable insights into the mechanisms underlying their anti‐TB effects. Furthermore, elucidating the safety profile of these nanocomposites is essential for their potential translation into clinical applications.

In summary, this research seeks to address the pressing need for new anti‐TB therapeutics by harnessing the synergistic properties of plant‐derived extracts and green‐synthesized nanoparticles. The integration of biological evaluation with comprehensive nanoparticle characterization is expected to advance our understanding of their therapeutic potential and pave the way for the development of effective anti‐TB strategies.

## 2 MATERIALS AND METHODS

### 2.1 Material

The astragalus and garlic samples were prepared from a local market and identified by a botanist in the herbarium of medicinal plants of the Medicinal Plants Research Center of the Zanjan Agricultural and Natural Resources Research Center. The plant samples were dried in a dark room at 20–28°C. Copper sulfate (CuSO4), sodium hydroxide (NaOH), and ethanol were provided from Sigma‐Aldrich (St. Louis, MO, USA) in pure form.

### 2.2 Extraction

To synthesize CuO nanoparticles (NPs) using green methods, garlic and astragalus extracts were utilized as both reducing and stabilizing agents. Initially, fresh garlic bulbs and astragalus roots were collected, washed thoroughly, and dried in shade. Subsequently, the dried plant materials were ground into fine powder using a grinder.

Next, the extraction of bioactive compounds from garlic and astragalus was performed. Briefly, 50 g of powdered garlic and astragalus was mixed separately with 500 mL of distilled water in a round‐bottom flask. The mixture was subjected to continuous stirring for 24 h at room temperature. Afterward, the resulting extracts were filtered using Whatman filter paper (Grade 1) to obtain clear aqueous solutions.

The obtained garlic and astragalus extracts were then utilized for the green synthesis of CuO nanoparticles. Details of the synthesis procedure, including reaction conditions and nanoparticle characterization, are described in subsequent sections.

The dried samples of garlic and astragalus were ground and powdered, and 100 g of each was suspended separately in 600 mL of hydroethanolic solution for 96 h at room temperature. The obtained solution was filtered from Soxhlet and then evaporated at 40°C. After that, the extracts were concentrated and filtered through a polyvinylidene fluoride (PVDF) membrane syringe filter (0.22 μm). The hydroalcoholic extract was concentrated under vacuum to obtain a dark brown extract and was kept at −20°C until it was used in the experiment (Daemi et al., 2019; Manzoureh & Farahpour, 2021).

### 2.3 Gas chromatography–mass spectrometry (GC–MS) analysis of garlic extract/analysis of extract components

The garlic extract was subjected to GC–MS analysis using a Shimadzu GC‐17A system that consisted of a gas chromatograph coupled with a mass spectrometer. This setup included an Agilent J&W capillary GC column (30 m, 0.32 mm, 1.00 μm) with 100% dimethylpolysiloxane stationary phase, along with a flame ionization detector (FID). Helium gas served as the carrier gas at a constant flow rate of 5 mL/min. The oven temperature was programmed to start at 70°C for 3 min, followed by a gradual increase to 300°C over 30 min. Mass spectra were obtained at 70 eV. A solvent delay of 3 min was implemented, resulting in a total GC/MS runtime of 25 min.

### 2.4 Synthesis of CuO nanoparticles

To synthesize CuO nanoparticles (NPs) using green methods, aqueous extracts of garlic and astragalus were utilized as both reducing and stabilizing agents. Initially, fresh garlic bulbs and astragalus roots were collected, washed thoroughly, and dried in shade. Subsequently, the dried plant materials were ground into fine powder using a grinder.

Next, the extraction of bioactive compounds from garlic and astragalus was performed. Briefly, 50 g of powdered garlic and astragalus was mixed separately with 500 mL of distilled water in a round‐bottom flask. The mixture was subjected to continuous stirring for 24 h at room temperature. Afterward, the resulting extracts were filtered using Whatman filter paper (Grade 1) to obtain clear aqueous solutions.

The obtained garlic and astragalus aqueous extracts were then utilized for the green synthesis of CuO nanoparticles. Details of the synthesis procedure, including reaction conditions and nanoparticle characterization, are described in subsequent sections.

### 2.5 Green synthesis of CuO NPs

One hundred milliliters of copper sulfate solution (0.1 M) was added to 50 mL of the prepared astragalus extract and placed on a magnetic stirrer for 1 h. The mixture's pH was raised to 9 by the addition of a 0.1 M NaOH solution. The solution was then placed in the microwave (MG48b, LG, South Korea) at 360°C for 5 min. The sample was cooled and then centrifuged at approximately 4472 g for 20 minutes. The change in the color of the extract from blue to dark yellow and turbidity indicated the production of nanoparticles. The resulting precipitates were collected, washed with distilled water, and then transferred into an oven at 72°C for 5 h to completely dry. The mixture was placed in a preheated furnace at 400°C for 60 min. The black fine product (NPs) was obtained and stored in an airtight glass container for further use.

### 2.6 Ultraviolet–visible (UV–Vis) analysis

Ultraviolet–visible spectroscopy (UV–Vis) was employed to track alterations in the solution's color caused by the absorption changes resulting from CuO NPs formation. The Shimadzu UV‐2550 spectrophotometer was used for characterizing the CuO NPs to study their kinetic behavior, with a scanning range from 200 to 800 nm. The data collected by the spectrophotometer were recorded and analyzed using the ‘UV Winlab’ software.

### 2.7 Fourier‐transform infrared (FTIR) spectroscopy

Fourier‐transform infrared (FTIR) spectra were obtained with a Bruker Tensor 27 FTIR spectrometer (Bruker, Germany) to analyze the chemical structure of the CuO NPs. Briefly, 2 mg of CuO NPs was incorporated into potassium bromide (KBr) pellets (100 mg) to create translucent sample disks. These sample disks were then analyzed in the spectral range of 400–4000 cm^−1^.

### 2.8 Polydispersity index, particle size, and zeta potential

At ambient temperature, a particle size analyzer based on dynamic light scattering (DLS) (Zetasizer Pro, Malvern Instruments Ltd., United Kingdom) was used to determine the polydispersity index (PDI), zeta potential (ZP), and particle size of the prepared nanoparticles.

### 2.9 X‐ray diffraction (XRD) analysis

The crystalline size and purity were assessed using a Siemens D5000 X‐ray diffraction (XRD) diffractometer (Aubrey, Texas, USA) with Cu‐Kα radiation (l = 1.5406 A) that was used for the X‐ray diffraction study in the scan range of 2*θ* from 10° to 80° at room temperature.

### 2.10 Field emission scanning electron microscopy (FE‐SEM) and energy‐dispersive X‐ray spectroscopy (EDS)

The surface morphology, elemental composition, and EDS analysis of the extract–CuO NPs were conducted with a field emission scanning electron microscope (FE‐SEM; TESCAN MIRA3, Czech Republic).

### 2.11 Transmission electron microscopy

To assess the morphology and precisely determine the size, a JEOL JSM 1200EX‐II transmission electron microscope, equipped with an electron diffraction pattern capability, was employed.

### 2.12 Cell viability

Cell viability was evaluated, as reported by other studies (Hwang et al., 2018). Briefly, peripheral blood mononuclear cells (PBMCs) were seeded in a 96‐well culture dish at a density of 2 × 105 cells per well. The cells were then treated with the help of nanoparticles diluted with a cell culture medium at different concentrations (2.5–50 μg/mL). The percentage of viable cells was investigated with the help of a cell counter, and cell viability (%) was calculated as live cells to total cells.

### 2.13 In vitro antibacterial activity

To prepare a suspension of *M. tuberculosis*, 2–3 colonies were picked up from a 4‐week culture in Löwenstein–Jensen medium containing glycerin, added into 4 mL of Middlebrook 7H9 culture media (Sigma‐Aldrich, St. Louis, MO, USA), and vortexed. The suspension was added into tubes after 30 min. The resulting turbidity was 3 × 10^8^ CFU (colony‐forming units)/mL, diluted, and a final concentration of 3 × 105 CFU/mL was obtained. Then, 10 mL of Middlebrook 7H9 culture media enriched with Oleic Acid Albumin Dextrose Catalase (Sigma‐Aldrich, St. Louis, MO, USA) was added to plates and 10 mL of garlic extract and astragalus extract–green‐synthesized CuO nanoparticles in dilutions of 100–0.39 μg/mL was added into wells. Then, 0.1 mL microbial suspension was added into each tube and submitted to 37°C. We determined both the minimum inhibitory concentration (MIC) and the minimum bactericidal concentration (MBC).

### 2.14 In vivo studies

University approved all the protocols for the care and treatment of the animals (IR.IAU.SHK.REC.1400.073). Fifty male Wistar rats with a weighting mean of 180–200 g were bought from the Faculty of Veterinary Medicine, University of Tehran (FVM‐UT, Iran). The rats were kept under standard temperature, light, and humidity conditions and had free access to water and food. As reported by other studies (Clark et al., 2015), tuberculosis was induced using the standard strain of H37Rv (Euro‐American lineage). The rats were grouped into four groups, including (1) Sham group with lack of tuberculosis, (2) rats with tuberculosis without treatment (negative control (NC)), (3) rats treated with 50 mg/kg nanoparticles in garlic extract (GN50) administered orally, and (4) rats treated with 100 mg/kg nanoparticles in garlic extract (GN50) administered orally. This study lasted for 21 days.

### 2.15 The expression level of ESAT‐6

To approve the produced antibody, polymerase chain reaction (PCR) was performed. In summary, a Bioner extraction kit was used to extract RNA from rat's whole blood by Total RNA Extraction Kit (Parstous, Tehran, Iran), and then complementary DNA (cDNA) was synthesized using Easy cDNA Synthesis Kit (Parstous, Tehran, Iran). The PCR method was employed to quantify the expression level of ESAT‐6. Primers included E6‐F (5′‐ACGAGATCTACAGAGCAGTGGAATTTC‐3′) and E6‐R (5′‐ ACGGGATCCTGCGAACATCCCAGTGA‐3′). Following electrophoresis, bacterial extracts were purified next to the protein by denaturing method to identify and confirm the band attributed to ESAT‐6 protein and transferred to the nitrocellulose membrane. Following incubation of the membrane with a dilution of 1/2000 His‐Tag antibody, its binding to the histidine tag was investigated using secondary antibodies bound to horseradish peroxidase (HRP) after the addition of 3,3 diaminobenzidine (DAB). The absorption at wavelength of 260 nm and also the appropriate ratio of 260/280 confirmed the purity of the extracted DNA. ESAT‐6 gene sequences were cut using corresponding endonuclease enzymes to create the desired fusion fragment and then ligated together by the enzyme ligase. The desired gene fragment was then cloned into the pQE‐30. Colon screening containing pqe30 was then carried out. The sediments from 1 mL of bacterial culture medium were electrophoresed on sodium dodecyl sulfate‐polyacrylamide gel electrophoresis (SDS‐PAGE). Based on the bands corresponding to the standard ESAT‐6 of *Mycobacterium* TB, the recombinant protein bands were identified. To identify and approve the ESAT‐6 protein, bacterial extracts were purified with the help of denaturing. Following incubation of membrane with dilution of 1/2000, His‐Tag antibody labeled was investigated.

### 2.16 Biochemical assessments

The rats were euthanized, and their lung tissues were preserved at −80°C for subsequent biochemical analyses. The samples were homogenized in ice‐cold normal saline (9 g/L) at a ratio of 1:9 (w/v). Afterward, the homogenates underwent centrifugation at approximately 1789 g for 10 minutes at 40°C. The biochemical analyses, including malondialdehyde (MDA), glutathione (GSH), superoxide dismutase (SOD), catalase (CAT), nitric oxide (NO), and thiol, were performed using commercial kits from Sigma‐Aldrich (St. Louis, MO, USA).

### 2.17 Histopathological analysis

Animals from each group were euthanized 21 days after cessation of treatment. Skin tissues were collected and promptly immersed in 10% neutral buffered formalin (NBF) (pH 7.26). Subsequently, the fixed tissue samples underwent processing, embedding in paraffin, and sectioning to achieve a thickness of 5 μm. Finally, the sections were stained with hematoxylin and eosin (H&E), following a previously established protocol (Cardiff et al., 2014).

### 2.18 Data analysis

Data normality was assessed using the Kolmogorov–Smirnov test in SPSS software (version 23). Subsequently, a one‐way analysis of variance (ANOVA) was performed since the data met the assumption of normality. To examine group differences, the Duncan post hoc test was applied, with statistical significance set at *p* < .05.

## 3 RESULTS AND DISCUSSION

### 3.1 Chemical profiling of garlic extract

**FIGURE 1:**
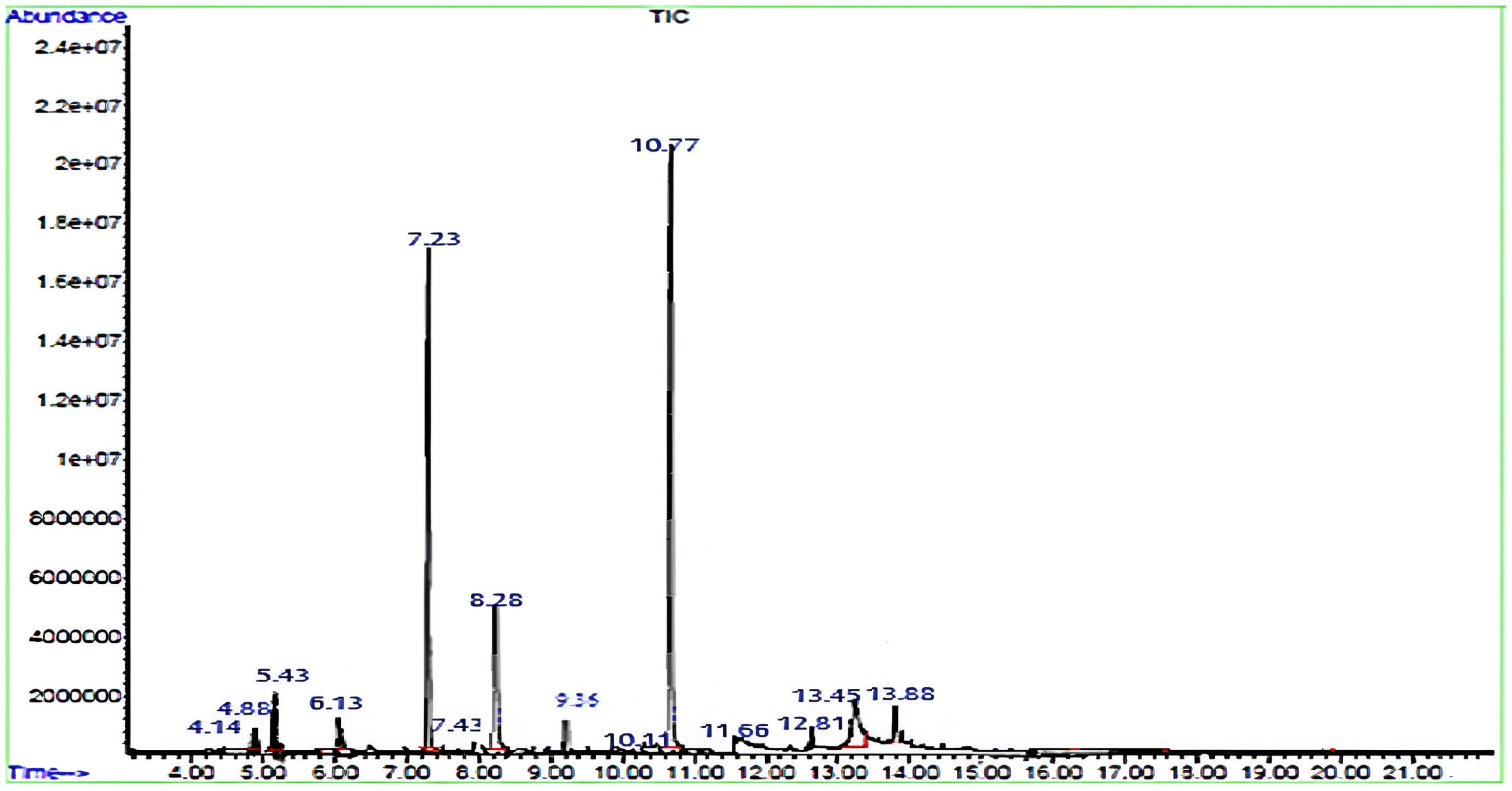
Gas chromatography–mass spectrometry (GC–MS) chromatogram of garlic extract.

The quantities of the compounds present in the aqueous garlic extract, determined through GC–MS analysis based on their respective retention times (see Figure 1), are presented in Table 1. All compounds contain sulfur, and the main ingredients of this garlic extract were di‐2‐propenyl trisulfide (36.22%), diallyl disulfide (27.52%), and methyl 2‐propenyl trisulfide (10.73%). The quantity of volatile compounds in garlic extract can be influenced by several factors that encompass genetic elements, such as garlic type and variety, as well as agricultural aspects including cultivation, crop management, and harvest conditions. Phytochemical analyses of the garlic extract were consistent with those used in previously described assays (Martínez‐Casas et al., 2017).

**TABLE 1:** The number of compounds in garlic extract.

No.	Component name	R.T (min)	%
1	1,3‐Dithiane	5.43	3.74
2	Dimethyl trisulfide	6.13	2.61
3	Diallyl disulfide	7.23	27.52
4	Diallyl disulfide (isomer)	7.43	0.85
5	Methyl 2‐propenyl trisulfide	8.28	10.73
6	3‐Vinyl‐1,2‐dithiocyclohex‐4‐ene	9.03	0.25
7	‐Amyl sec‐butyl disulfiden	9.36	2.53
8	3‐Vinyl‐1,2‐dithiocyclohex‐5‐ene	9.74	0.23
9	di‐2‐propenyl trisulfide	10.77	36.22
10	Pyrogallol	11.66	0.97
11	Hexathiane	13.45	2.83
12	Diallyl tetrasulfide	13.88	2.51

### 3.2 Ultraviolet (UV) analysis of garlic nanoparticles

**FIGURE 2:**
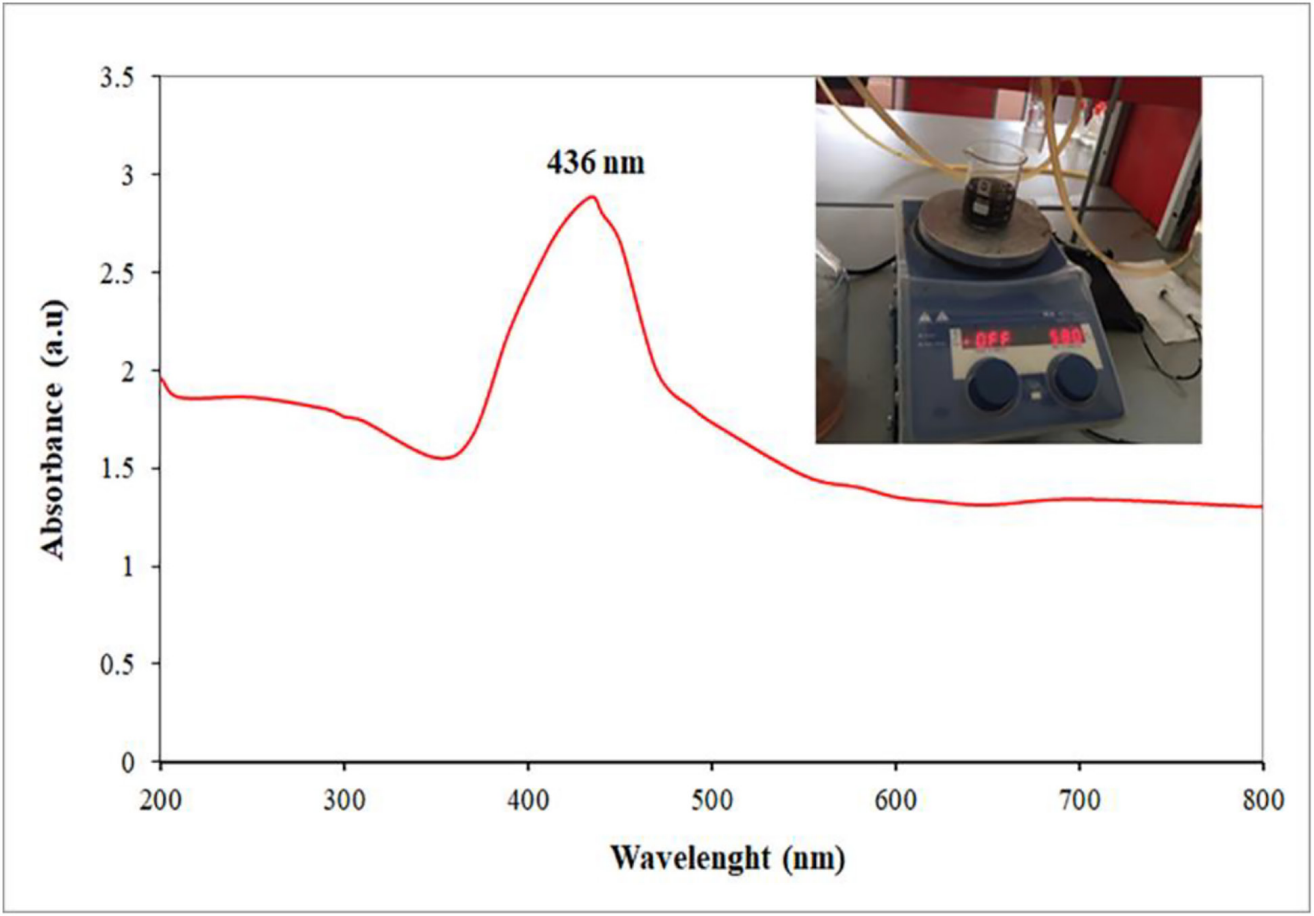
Ultraviolet‐visible (UV–vis) absorption spectra of the prepared solutions of CuO NPs.

Figure 2 displays the ultraviolet–visible (UV–vis) absorption spectra of CuNPs produced using the plant's aqueous extract. This extract comprises various compounds, including phenolics, tannins, saponins, proteins, flavonoids, glycosides, and polyphenols, serving as both reducing and stabilizing agents (Liu et al., 2020). These materials reduce Cu^2+^ to Cu^0^. The progress of the reaction can be observed by a visual color change from light blue to dark brown due to the excitation of surface plasmon vibrations in the CuO NPs (Liu et al., 2020). Copper salt when combined with plant extract forms a polyphenolic compound with Cu^2+^ ions. Further reduction leads to the conversion of Cu^2+^ to Cu^0^ NPs. The maximum absorption peak was obtained at the wavelength of 436 nm, which indicates the surface plasmon resonance (SPR) for CuO NPs. This peak arises from the surface plasmon absorption of the metal oxide. In metal oxide nanoparticles, surface plasmon absorption occurs because of the collective oscillation of unbound conduction band electrons that become excited when exposed to incident electromagnetic radiation. This resonance phenomenon takes place when the wavelength of the incident light significantly exceeds the diameter of the particles (Veisi et al., 2021).

### 3.3 Fourier‐transform infrared (FTIR) spectroscopic analysis of garlic nanoparticles

**FIGURE 3:**
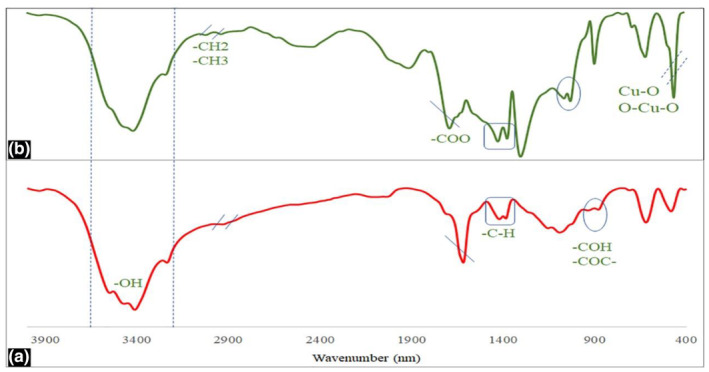
The FTIR spectrum of (a) astragalus extract and (b) astragalus extract–green‐synthesized CuO NPs.

Figure 3 depicts the FTIR spectrum of the astragalus extract and astragalus extract–green‐synthesized CuO NPs. The band observed at 3440 cm^−1^ is associated with stretching vibrations of surface hydroxyl groups, absorbed water, and/or numerous hydroxyl groups in the astragalus extract used for the synthesis of nanoparticles. The stretching vibrations of various C–H groups at 2785 cm^−1^ and 2843 cm^−1^ and the bands at 1700 cm^−1^ to 1450 cm^−1^ regions can be assigned to the numerous stretching and bending vibrations of C=C and C–H groups. The prominent absorption peak observed at 1742 cm^−1^ corresponds to the stretching vibration of the carbonyl group present in the astragalus extract. The characteristics of bands in the CuO NPs appeared as strong stretching vibrations of Cu–O and O–Cu–O bonds at 532 cm^−1^ (Ul‐Hamid et al., 2022).

### 3.4 X‐ray diffraction (XRD) analysis of garlic nanoparticles

**FIGURE 4:**
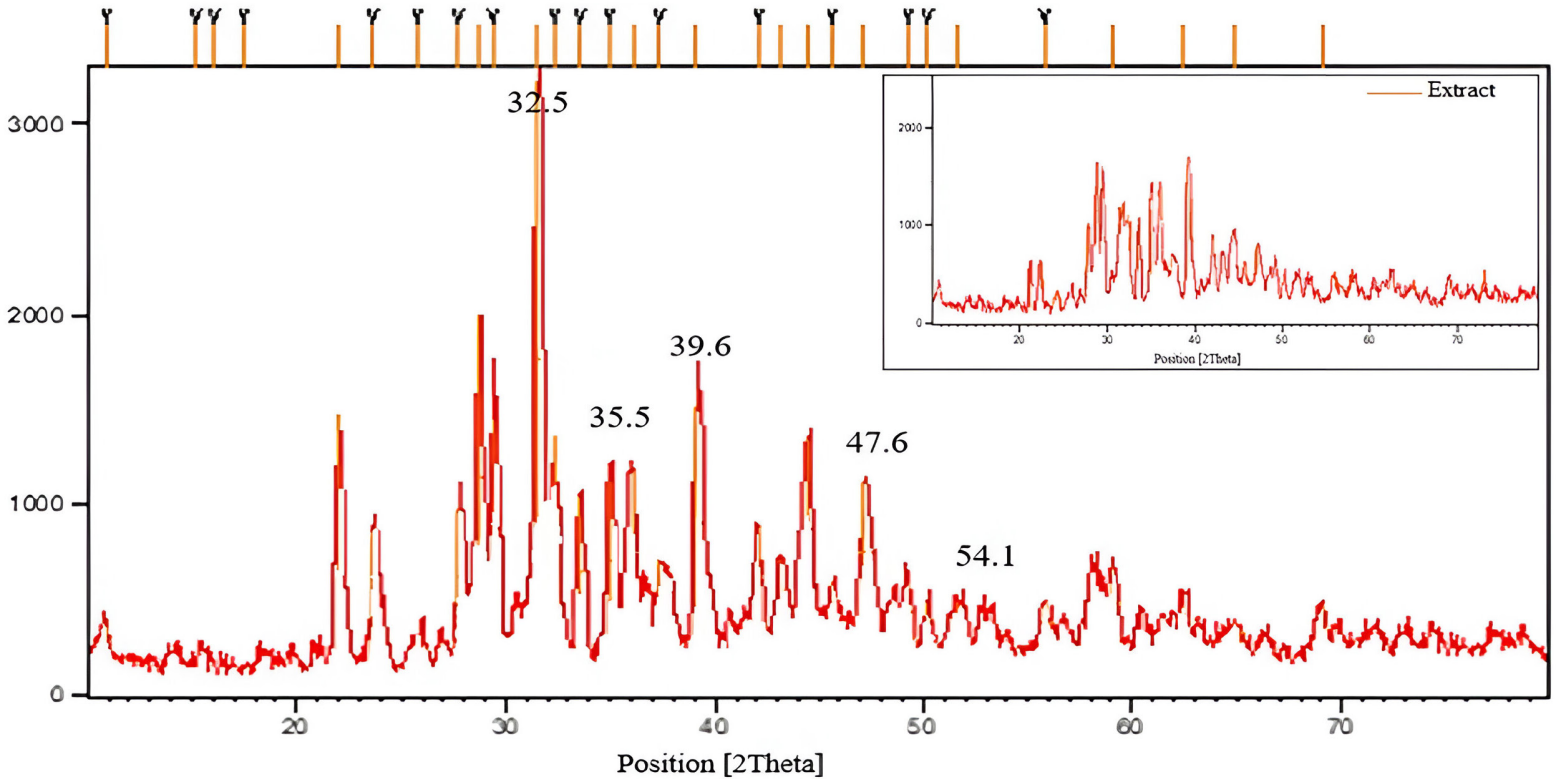
X‐ray diffraction (XRD) pattern of astragalus extract and astragalus extract–green‐synthesized CuO NPs.

X‐ray diffraction analysis was used to determine the orientation of CuO nanoparticles (Figure 4). The resulting XRD spectrum has 11 peaks, with two index peaks at 35.51 and 39.15 assigned to the (002) and (111) diffractions of CuO crystals, respectively. The angles of the peaks are given in Table 2. Using standard spectra (JCPDS (Joint Committee on Powder Diffraction Standards); Card No. 0937‐045) and comparing them, it can be concluded that the intended material is CuO nanoparticles, and their structure is monoclinic. The size of the Cu NPs was calculated from Debye–Scherr's equation (Equation (1))

(1) $$d=\frac{\mathrm{k\lambda}}{\beta \;\cos\theta }$$

where *d* is the average particle size (nm), *k* is the shape factor (0.9), *λ* is the X‐ray radiation wavelength (1.54060 A°) for Cu Kα, *β* is the full width at half maximum (FWHM) of the peak, and *θ* is peak Bragg's angle and ranged from 53.36 to 78.66 nm for the CuO‐doped extract, as shown in Figure 4.

**TABLE 2:** The various peaks in the XRD pattern of A–CuO nanoparticles.

Plane	(110)	(002)	(111)	(202)	(020)	(202)	(113)	(311)	(113)	(311)	(004)
(2θ)o	32.57	35.51	39.15	47.61	54.18	58.13	61.56	66.60	68.39	72.73	75.25

### 3.5 The zeta potential and dimensions of the nanoparticles produced

**FIGURE 5:**
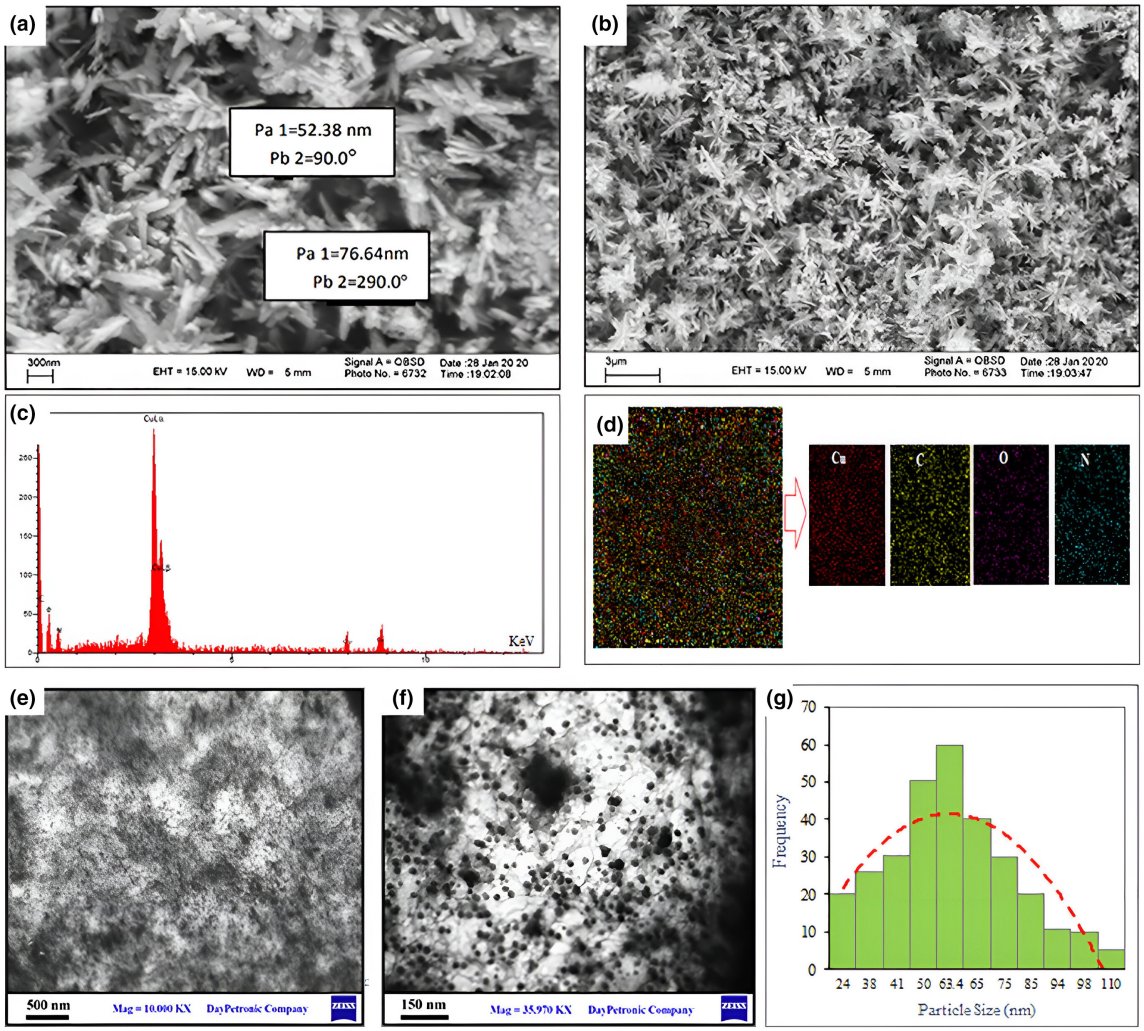
(a, b) Field emission scanning electron microscopy (FESEM), (c) EDS, and (d) dot mapping, (e, f) TEM, and (g) PSD of A–CuO NPs.

Under the given synthesis conditions, the prepared CuO NPs exhibited a PDI of 0.243 and a zeta potential of +32 mV. The value of zeta potential indicates that positively charged groups surround the nanoparticles and cause stability. The average particle size distribution (PSD), as illustrated in Figure 5, was measured at 52 nm. This finding suggests that the astragalus extract effectively produced CuO NPs with reduced particle size and enhanced stability, likely attributed to the elevated zeta potential. Furthermore, the presence of a single peak in the data indicated that the quality of the synthesized astralagus (A)–CuO NPs was of high standard.

### 3.6 The FE‐SEM and TEM images of garlic nanoparticles

**FIGURE 6:**
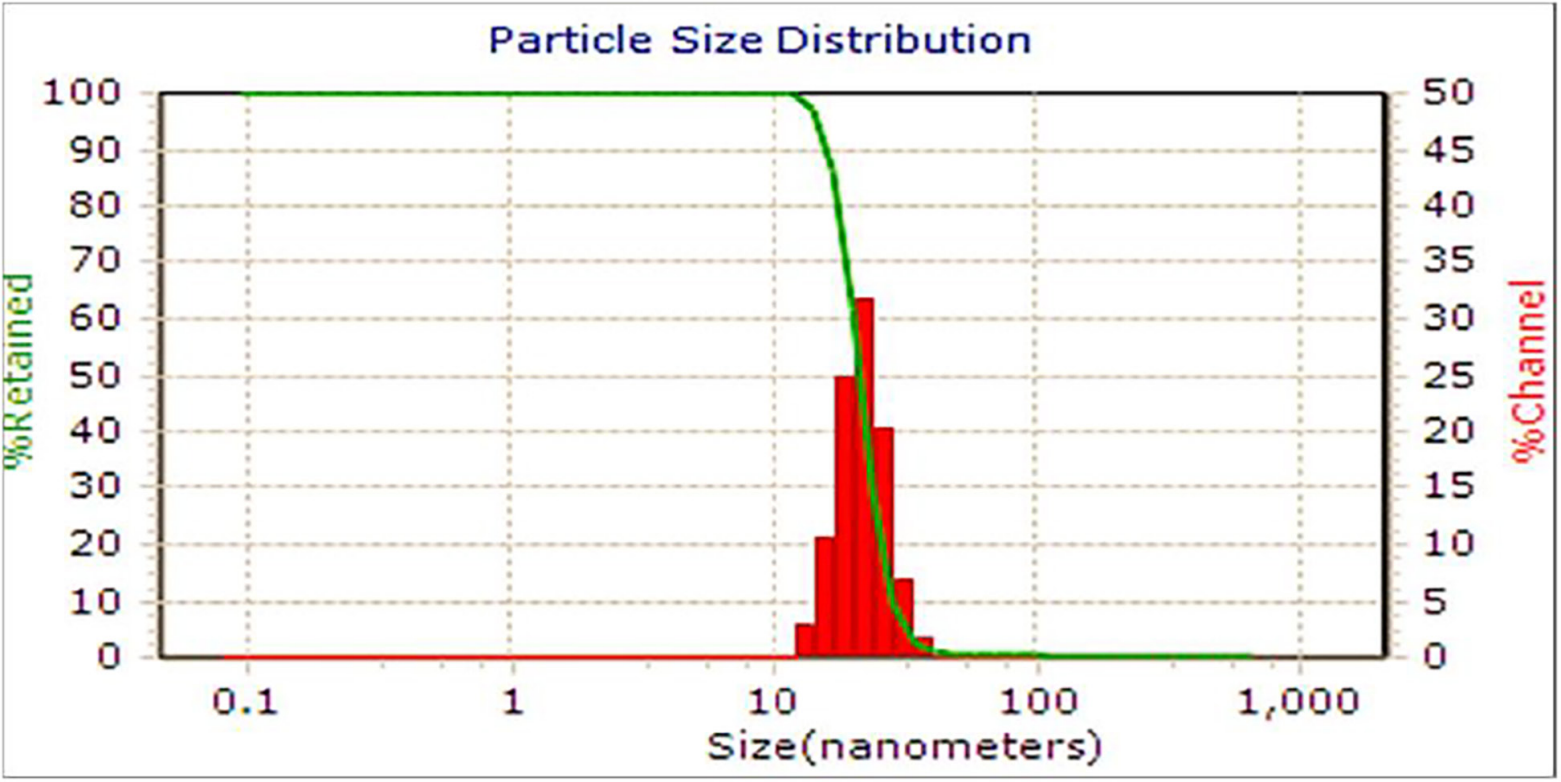
The particle size diagram of the synthesized A–CuO NPs.

The morphology of the A–CuO NPs was characterized by FE‐SEM and TEM imaging (Figure 5). The synthesized A–CuO exhibited a three‐dimensional (3D) structure, spherical structures, and dense agglomeration in Figure 5a,b. The particle size analysis results indicated an average size of 50 nm, as depicted in Figure 6. Additionally, the EDS spectrometer confirmed the presence of signals associated with both copper and oxygen elements, as shown in Figure 5c. The EDS result also showed that prepared A–CuO NPs contain 64.6% of copper (Cu), 23% of oxygen (O), 10.1% carbon (C), and 2.3% of nitrogen (N). Also, the elemental mapping image of A–CuO NPs (Figure 5d) showed that the Cu element is the main component dispersed throughout the substrate. TEM analysis was accomplished to identify the morphology of the formed A–CuO NPs. TEM images of the fabricated NPs at obtained synthesis conditions showed that the particles were monodispersed and spherical and thermodynamically stable with minimum surface energy, which corresponds to a high value of zeta potential (Figure 5e,f) (Pansambal et al., 2017). Moreover, histogram of CuO NPs obtained by Nano Measurer supports the SEM and TEM observations showing 63.41 nm mean particle size as depicted in Figure 5f to be related to the size of CuO NPs in the astragalus extract.

### 3.7 Cell viability analysis of garlic nanoparticles

**FIGURE 7:**
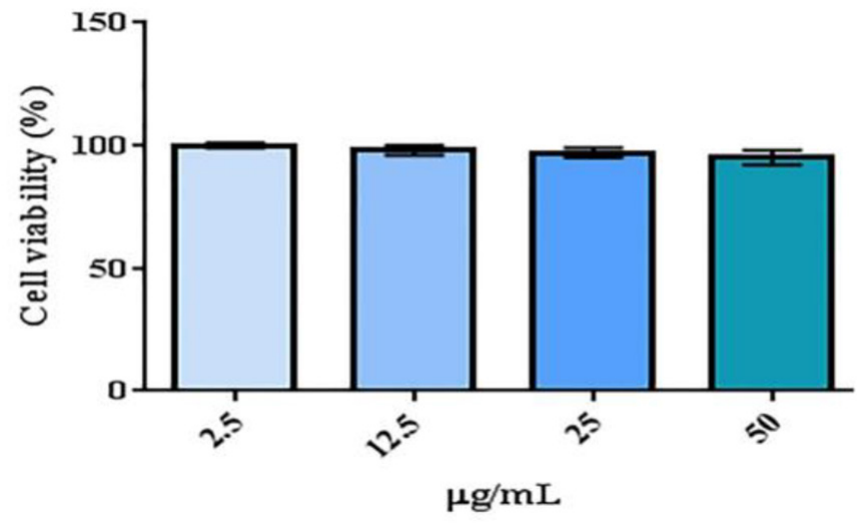
The results for cell viability of the different concentrations of garlic and nanoparticles extract.

Figure 7 depicts the results for cell viability of the prepared nanoparticles. The results showed that the nanoparticles did not exhibit any toxicity. The outcomes did not reveal noteworthy distinctions among the various groups (*p* = .815). The results are in contrast with the results reported by others on the toxicity of copper nanoparticles (Bugata et al., 2019; Naz et al., 2020). These findings align with those of previous research on the safety of copper nanoparticles synthesized using plant extracts (Bhattacharya et al., 2019; Sulaiman et al., 2018). Thus, green synthesis helps in the safety of the prepared nanoparticles.

### 3.8 Antibacterial activity of garlic nanoparticles

**FIGURE 8:**
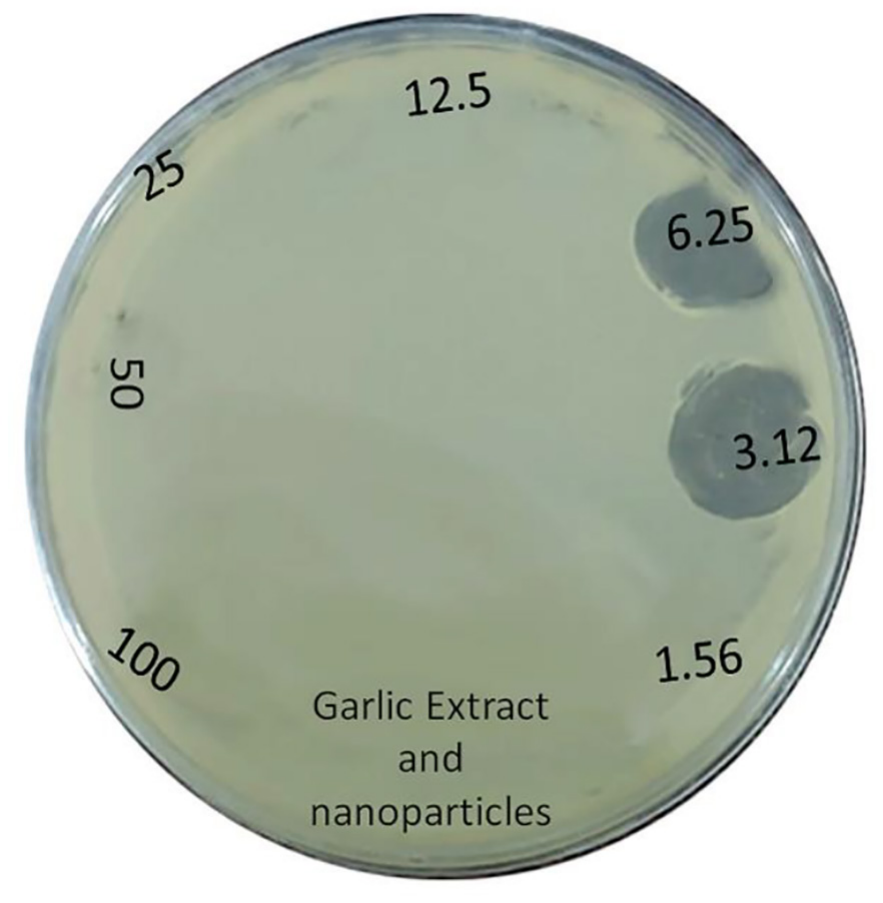
The results for antibacterial activities of dilutions of garlic and nanoparticles extract.

Table 3 presents the outcomes of the nanoparticles' antibacterial properties. The results showed that the nanoparticles in lower dilutions exhibited greater antibacterial activities. Figure 8 validates the antibacterial efficacy of the nanoparticles using the diameter zone method.

**TABLE 3:** Antibacterial activities of the nanoparticles.

Tests	100%	50%	25%	12.5%	6.25%	3.12%	1.56%	0.78%	0.39%	Control
MIC	−	−	−	−	+	+	+	+	+	−
MBC	−	−	−	−	+	+	+	+	+	−

Abbreviations: −, Absence of antibacterial activities; +, Presence of antibacterial activities; MBC, Minimum bactericidal concentration; MIC, Minimum inhibitory concentration.

The results show antibacterial activities of the nanoparticles. Similarly, recent studies reveal that *Cocculus Hirsutus*, *Piper nigrum*, and *Phoenix dactylifera* present a valuable resource for the green synthesis of CuNPs, showcasing significant in vitro antioxidant, antibacterial, and antidiabetic properties (Allemailem et al., 2022; Ameena et al., 2022; Kiranmayee et al., 2023).

The outcomes concerning the antibacterial properties of CuO nanoparticles align with findings from prior research (Sathiyavimal et al., 2021; Vasantharaj et al., 2019). CuO nanoparticles interact with the plasma membrane of bacteria, lead to holes in the membrane, and lyse bacterial cell (Concha‐Guerrero et al., 2015). The released Cu^2+^ ions also interact with negative charge of the cell wall and membrane and cause denaturation and alteration in the membrane proteins (Yoon et al., 2007). Copper ions inside the bacterial cell produce reactive oxygen species (ROS) and cause changes in cellular signaling and interfere with the nucleic acid, which results in an altered helical structure (Gunawan et al., 2011). In the current study, the nanoparticles exhibited antibacterial activities against *M. tuberculosis* and its exact mechanism needs future investigations.

### 3.9 Effects of garlic extract and the nanoparticles on induction of disease

The tuberculin diagnostic tests showed that oral gavage of 50 mg/kg of the mixture of the garlic extract and nanoparticles could not prevent the induction of disease and tuberculosis was seen in all the rats. However, tuberculosis was seen only in 20.00% of rats treated with 100 mg/kg of the mixture of the garlic extract and nanoparticles. The results show that the daily administration of garlic extract and the nanoparticles significantly prevents diseases. There are no studies investigating the effects of CuO nanoparticles synthesized with the help of medicinal plants and incorporated with plant extracts. However, the effects of garlic extract with the produced nanoparticles could be attributed to their effects in improving antioxidant activities and preventing damage to lungs, as will be discussed. In addition, the antibacterial activities of the nanoparticles may prevent the growth and replication of bacteria.

### 3.10 Identification and confirmation of recombinant proteins of garlic nanoparticles

**FIGURE 9:**
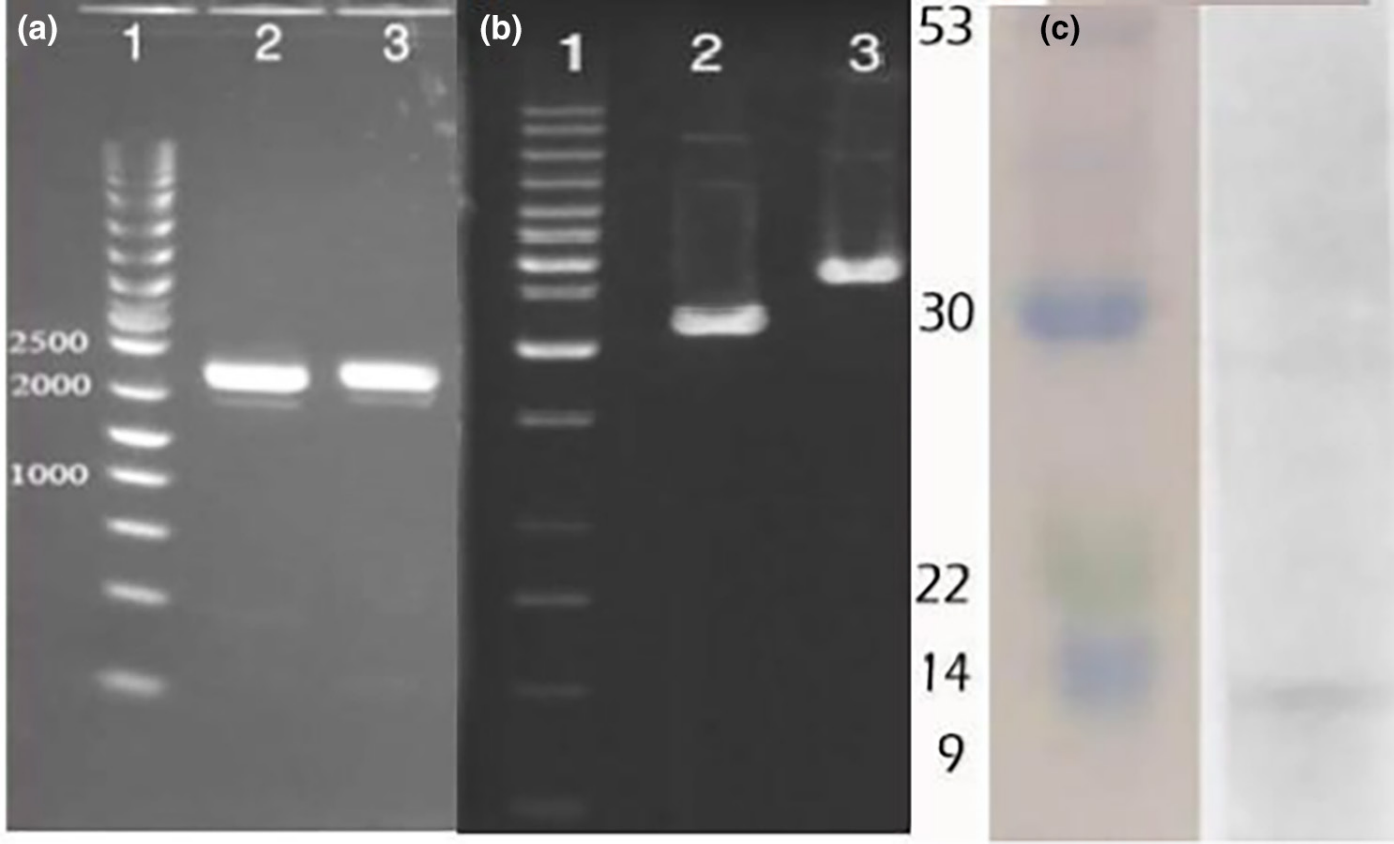
The results for molecular section. PCR product. (a) Transformed colony of pqe30 (b) and Western blotting (c).

Identification and confirmation of recombinant proteins were performed by Western blotting assessment method. The PCR test confirmed sequences for protein gene (Figure 9a), and the target gene fragment was cloned with pqe30 vector (Figure 9b). A comparison of the Western blot‐stained membrane and its equivalent SDS‐PAGE gel showed that only the proteins detected on the membrane were associated with the recombinant proteins and histidinically labeled in the post‐induction column and the post‐purification column (Figure 9c). ESAT protein is one of the secretory antigens of bacteria and it is the first protein produced at the early stage of infection, owing to the cellular and humoral immune responses (Samten et al., 2011). Song et al. (2014) detected the ESAT‐6 antigen in a cerebrospinal sample of patients with tuberclosis disease using the enzyme‐linked immunosorbent assay (ELISA) method with sensitivity and specificity of 82% and 92%, respectively. Recombinant *Mycobacterium tuberculosis* proteins in Iran have been cloned successfully and expressed separately or together too and used diagnostically (Xu et al., 2012).

### 3.11 Biochemical analysis of garlic nanoparticles

**FIGURE 10:**
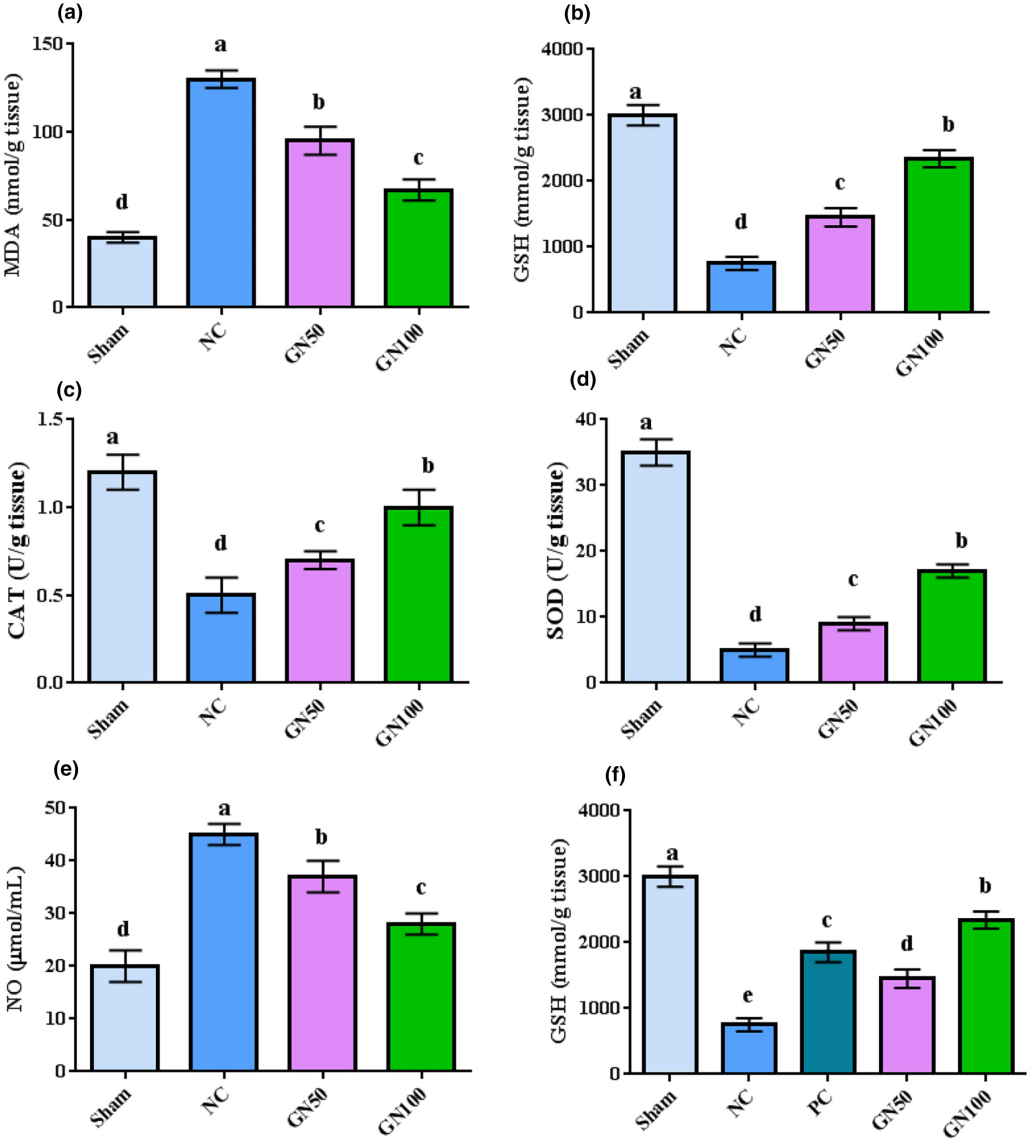
The results for the effects of garlic extract and nanoparticles on antioxidant parameters. Different superscripts show significant differences between groups. Sham, sham group; NC, negative control; GN50, garlic extract + nanoparticles in level of 50 mg/kg, and GN50, garlic extract + nanoparticles in level of 100 mg/kg.

Figure 10 depicts the results for the effects of garlic extract and nanoparticles on the antioxidant status. The results showed that infection with *M. tuberculosis* significantly increased MDA and NO while decreasing GSH, CAT, SOD, and thiol, as sham was compared with NC (*p* = .0001). The rats treated with garlic and nanoparticles at a level of 50 mg/kg showed lower MDA and NO and higher GSH, CAT, SOD, and thiol compared with negative control (*p* = .0001). The rats administered with 100 mg/kg garlic and nanoparticles showed the highest GSH, CAT, SOD, and thiol compared with those of other groups excluding the control sham.

The results for the effects of infection with *M. tuberculosis* on MDA and NO while decreasing GSH, CAT, SOD, and thiol are in agreement with those of previous studies (Farahpour & Ghayour, 2014; Gravier‐Hernández & Gil‐del Valle, 2018; Khan & Ali, 2018; Meca et al., 2021). SOD, CAT, and GSH remove reactive oxygen species before DNA, proteins, and/or lipids attack. SOD can be converted to hydrogen peroxide (H_2_O_2_). Indeed, antioxidants inhibit tuberculosis manifestations and prevent tissue degradation (Jeremiah et al., 2021). Based on the findings, infection with *M. tuberculosis* increases MDA and NO while decreasing the concentration and activities of antioxidant and non‐antioxidant enzymes. Under infection with *M. tuberculosis*, macrophages undergo respiratory burst after contact with the bacterium and generate reactive oxygen species and DNA damage (Chen et al., 2012). Seemingly, the increased reactive oxygen species causes to increase MDA and NO. The increased ROS and MDA may increase the release of antioxidants and result in decreased amounts. The results showed that the administration of garlic and nanoparticles in higher amounts could improve antioxidant status via increasing enzymes and decreasing MDA and NO. The results are in agreement with the results reported for the antioxidant properties of CuO nanoparticles synthesized with the help of plant extracts (Ijaz et al., 2017; Rehana et al., 2017; Thakar et al., 2022). Metal chelation by polyphenolic compounds is extensively considered as another mechanism of their antioxidant activity (Sorbiun et al., 2018). Phenolic compounds are antioxidants and quench oxygen‐derived free radicals by donating hydrogen atom or an electron to the free radicals (Sorbiun et al., 2018). It was reported that phytochemicals in plant extracts may enhance antioxidant activity of copper nanoparticles (Wu et al., 2020). Our findings showed that copper nanoparticles prepared with the help of plant extracts have antioxidant activity. The antioxidant activity of the nanoparticles helps to fight against pathogens and prevent the progression of diseases. Greater antioxidant activity in 100 mg/kg compared with that in 50 mg/kg could be attributed to more active compounds in garlic and the prepared nanoparticles.

### 3.12 Effects of garlic extract and nanoparticles on the structural damage in a rat's lung

**FIGURE 11:**
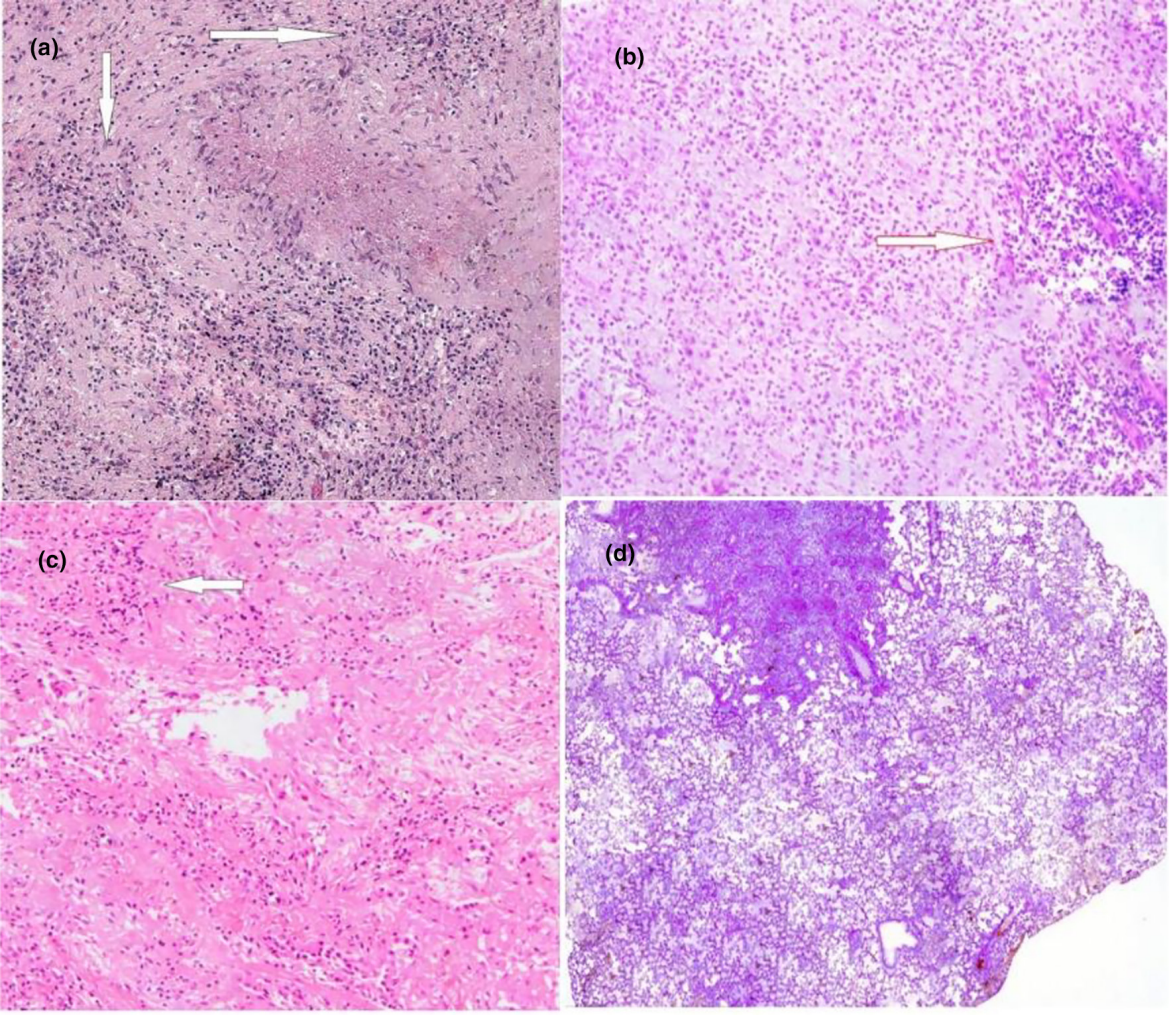
Stained microscopic sections (magnification 100×) of the effect of garlic extract and nanoparticles on the structural damage in rat lungs. (a) Sham (control positive): the sham group did not receive any garlic extract + nanoparticles, tissue necrosis and granulomatous necrosis were clearly identified. (b) Garlic extract + nanoparticles in level of 100 mg/kg: the lung tissue of mice suffering from tuberculosis, which was exposed to 100 mg/kg of garlic extract+copper nanoparticles, the destruction of the lung tissue has decreased. (c) Garlic extract + nanoparticles in level of 50 mg/kg. (d) Negative control: without receiving any garlic extract + nanoparticles.

Figure 11 depicts the results for H&E staining. The results showed that induction of disease was completely conducted, and animals were involved with disease. Compared with intact animals, other animals showed damage to lung tissue. However, the administration of garlic and nanoparticles could significantly alleviate negative effects of tuberculosis on lung tissue and necrosis. Nanoparticles exhibited their effects in a dose‐dependent manner. Decreased negative effects of garlic and nanoparticles on lung tissue could be attributed to antibacterial activities and antioxidant properties of garlic extract and nanoparticles. In sum, nanoparticles prevent lung damage via antioxidant and antibacterial activities.

## 4 CONCLUSIONS

In sum, the mixture of garlic extract and nanoparticles at a concentration of 100 mg/kg body weight of rats inhibited tuberculosis infection. It exhibited antibacterial activity and antioxidant properties in lung tissue and also prevented lung damage. The results are promising and show that garlic extract and nanoparticles can be utilized to prevent tuberculosis. This study was conducted on rats and the results cannot be used for humans that are a major limitation.

## AUTHOR CONTRIBUTIONS

Data curation; investigation; methodology; resources; validation; visualization; writing – original draft: ZZ. Conceptualization; data curation; formal analysis; funding acquisition; investigation; methodology; project administration; supervision; validation; writing – review and editing: ET, NAS. Conceptualization; formal analysis; methodology; project administration; supervision; validation; writing – review and editing: AMG.

## FUNDING INFORMATION

This research did not receive any specific grant from funding agencies in the public, commercial, or not‐for‐profit sectors.

## CONFLICT OF INTEREST STATEMENT

The authors declare that there is no conflict of interest.

## ETHICS STATEMENT

The author confirms that the ethical policies of the journal as noted on the journal's author guidelines page have been adhered to and the appropriate Ethical Review Committee's approval has been received.

## Data Availability

The data used to support the findings of this study are available from the corresponding author upon request.
